# Susceptibility of Fall Armyworms (*Spodoptera frugiperda* J.E.) from Mexico and Puerto Rico to Bt Proteins

**DOI:** 10.3390/insects11120831

**Published:** 2020-11-26

**Authors:** Rebeca Gutierrez-Moreno, David Mota-Sanchez, Carlos A. Blanco, Desmi Chandrasena, Christina Difonzo, Jeffrey Conner, Graham Head, Kristina Berman, John Wise

**Affiliations:** 1 Department of Entomology, Michigan State University, 1129 Farm Lane, East Lansing, MI 48824, USA; gutie131@msu.edu (R.G.-M.); difonzo@msu.edu (C.D.); wisejohn@cns.msu.edu (J.W.); 2 Department of Biology, University of New Mexico, Albuquerque, NM 87131, USA; carlos.blanco1206@gmail.com; 3 Corteva Agriscience, Johnston, IA 50131, USA; desmi.chandrasena@corteva.com; 4 Department of Plant Biology, W. K. Kellogg Biological Station, Michigan State University, Hickory Corners, MI 49060, USA; connerj@msu.edu; 5 Bayer U.S.-Crop Science, Chesterfield, MO 63017, USA; graham.head@bayer.com (G.H.); kristina.berman@bayer.com (K.B.)

**Keywords:** *Spodoptera frugiperda*, resistance, Bt corn, bioassays, LC_50_, EC_50_

## Abstract

**Simple Summary:**

Fall armyworms from Mexico are susceptible to four *Bacillus thuringiensis* (Bt) proteins, while those from Puerto Rico have field-evolved resistance to Cry1F and Cry1Ac and are susceptible to Cry2Ab2 and Cry1A.105. Implications for fall armyworm management are discussed.

**Abstract:**

Fall armyworm is one of the main pests of conventional and *Bacillus thuringiensis* (Bt) corn in many countries in the Americas, Africa, Asia and in Australia. We conducted diet-overlay bioassays to determine the status of susceptibility to four Bt proteins (Cry1A.105, Cry2Ab2, Cry1F and Cry1Ac) in three different populations of fall armyworm from Mexico, and one population from Puerto Rico. Bioassays showed that fall armyworms from Puerto Rico were resistant to Cry1F with a resistance ratio _50_ (RR_50_) higher than 10,000 ng/cm^2^ and to Cry1Ac with a RR_50_ = 12.2 ng/cm^2^, displaying the highest median lethal concentration (LC_50_) values to all Bt proteins tested. The effective concentration _50_ (EC_50_) values further confirmed the loss of susceptibility to Cry1F and Cry1Ac in this population. However, LC_50_ and EC_50_ results with Cry1A.105 and Cry2Ab2 revealed that fall armyworm from Puerto Rico remained largely susceptible to these two proteins. The Mexican populations were highly susceptible to all the Bt proteins tested and displayed the lowest LC_50_ and EC_50_ values to all Bt proteins. Our results suggest that Cry1F and Cry1Ac resistance is stable in fall armyworm from Puerto Rico. However, this population remains susceptible to Cry1A.105 and Cry2Ab2. Results with Mexican fall armyworms suggest that possible deployment of Bt corn in Mexico will not be immediately challenged by Bt-resistant genes in those regions.

## 1. Introduction

Fall armyworm *Spodoptera frugiperda* (J.E. Smith) (Lepidoptera: Noctuidae) is native to the Americas and is a major pest in the southern United States and Latin America, affecting important crops such as corn, rice, sorghum, and cotton [1,2]. On corn, larvae feed mainly on foliage, but occasionally feed in the ear [3,4]. Management of fall armyworm is complicated by its outstanding migratory capacity, as it can move long distances between countries and even regions [5]. In 2016, it was first detected in West Africa, and it spread to 28 African countries within a year of detection [6]. By 2020, fall armyworm was officially confirmed in Yemen, several countries in Southeast Asia, and Australia, threatening the production of not only corn, but rice, sorghum, and other crops [7,8].

Fall armyworm management is also difficult because of its resistance to both foliar insecticides [9,10,11,12], and genetically engineered (GE) corn producing insecticidal proteins from *Bacillus thuringiensis* (Berliner) (Bt) [13,14,15,16,17,18,19,20,21,22]. In most Latin American countries, fall armyworm is considered the most important pest in both conventional and Bt corn production [23]. In many cases, additional synthetic insecticide sprays have been required to achieve satisfactory control levels in GE crops [23,24].

Fall armyworm populations have been described to display inherent low susceptibility to the Bt proteins Cry1Ab and Cry1Ac [19,25,26]. Yet, the first report of unexpectedly high damage in Bt corn by fall armyworm occurred in Puerto Rico, 10 years after the introduction of Bt maize (TC1507) producing Cry1F [13,14]. Since then, field-evolved resistance cases have been reported in Puerto Rico to Cry1F, Cry1Ac and Cry1Ab [13,14,15]; in Brazil to Cry1F and Cry1Ab [16,17,18]; in the southeastern United States to Cry1A.105 and Cry1F [19,21]; and in Argentina to Cry1F [22].

Fall armyworm is the main corn pest in Puerto Rico. Since pyramided Bt maize production on this island is mainly grown for hybrid seed increase and research, acceptance of pest damage is very low, which has led to a high number of insecticide applications to control fall armyworm [10,12,23]. This means that fall armyworm is under strong selection pressure, which has caused resistance to evolve to different insecticide modes of action [12], in addition to the reported resistance to Cry1F and Cry1Ac [13,14,27]. Factors such as weather conditions, relative isolation and continuous generation of fall armyworm contributed to the first reported evolution of resistance to Bt proteins in Puerto Rico [15]. Although weather conditions are variable, relative isolation and continuous generation are a constant on this island. Furthermore, pyramided corn hybrids grown in Puerto Rico still contain Cry1F, with the addition of Cry1A.105 and Cry2Ab2. Since there have been reports of cross-resistance between Cry1 proteins in South America [28], it is important to monitor the susceptibility status of fall armyworms from Mexico and Puerto Rico to Bt corn because of the potential migration of resistant fall armyworms to Mexico.

In Mexico, fall armyworm is the hardest pest to manage in most corn producing regions, and control relies heavily on synthetic insecticide sprays and Bt corn technology is not commercially available [12,23,29]. Farmers spray insecticides two or three times per crop cycle to control fall armyworm and other insect pests, using mostly organophosphates and pyrethroids [23,29]. However, some growers report that they spray up to 12 times to control fall armyworm (Mota-Sanchez D, unpublished). The use of synthetic insecticides as the sole control measure can lead to problems such as the evolution of resistance to the commonly used chemistries, loss of arthropod biodiversity by affecting non-target organisms, and environmental pollution. However, the heterogeneity of corn production in Mexico, which ranges from small subsistence farms to large commercial enterprises, complicates the application of effective integrated pest management (IPM) strategies throughout the country [29].

Monitoring the susceptibility of target pests to control tools is a key component in IPM programs, as it allows for changes in management tactics before resistance is fixed in a population [30]. Knowing the level of susceptibility of a pest to a pesticide before the technology is deployed in the field can help strategically plan its application. Fall armyworm populations from different regions in Latin America have displayed variability in their susceptibility to Bt proteins [31]. Therefore, the objective of this study was to determine and compare the susceptibility status of different populations of fall armyworm to Cry1F, Cry1Ac, Cry1A.105 and Cry2Ab2. These populations were from three different locations in Mexico, where the larvae have not been exposed to Bt corn, and from one location in Puerto Rico, where the larvae have been in contact with Bt expressing corn for over a decade.

## 2. Materials and Methods

### 2.1. Field and Laboratory Fall Armyworm Populations

Fall armyworm larvae from Mexico were collected during summer 2015 in cornfields from the Northwest (Los Mochis, Sinaloa (Sin)), West Central (San Martin de Hidalgo, Jalisco (Jal)) and Southwest (San Pablo Huitzo and San Lorenzo Cacaotepec Etla, Oaxaca (Oax)). San Pablo Huitzo and San Lorenzo Cacaotepec are located about 30 km apart from each other. The corn fields where fall armyworms were collected in Oaxaca share weather conditions and agronomical practices, since they belong to small subsistence farmers. Therefore, fall armyworms were pooled together and their progeny (F1–F3) were used in the bioassays.

Larvae from Puerto Rico (PR) were collected in June to November 2016. Bayer Crop Science (formerly Monsanto, St. Louis, MO, USA) provided a fall armyworm susceptible laboratory (Sus) population from their rearing facilities in Union City, Tennessee.

For each population, a minimum of 200 fourth- and fifth-instar larvae were collected directly from maize plants. Larvae were placed in 10 mL plastic cups with 4–6 mL of artificial diet (Southland Products Incorporated: fall armyworm diet), and sent to our laboratory at Michigan State University, East Lansing, Michigan, United States.

### 2.2. Insect Rearing

Larvae were held at 27 ± 1 °C and a photoperiod of 16:8(L:D) h. Insect development was recorded every other day. When pupae changed colors from orange-red to dark red, they were placed in collapsible insect cages (Bioquip, Rancho Dominguez, CA, USA) in groups of 40 individuals, to provide room for emerging adults to fly and mate. A sponge with a solution of 10% sucrose was placed inside the cages and replenished every other day. After three days of adult emergence, adults from each cage were placed in 1 L paper brown bags with the same food source. Moths were transferred to a new paper bag every other day. The eggs masses laid in the paper bag were separated and placed in a plastic container with moist paper towels. Neonate larvae were transferred to bioassay trays (Frontier Agricultural Sciences, Newark, DE, USA) with 1 mL of artificial diet per well.

### 2.3. Bt Proteins

Corteva Agriscience (formerly Dow AgroSciences LLC, Indianapolis, IN, USA) provided lyophilized Cry1F and Cry1Ac proteins and 10 mM 3-(cyclohexylamino)-1-propane sulfonic acid (CAPS buffer), diluted in deionized water, pH 10.5. Proteins were kept in a desiccator (Fisher Scientific rectangular Desi-Vac Container™), both proteins and buffer stored in a refrigerator at 4 °C.

Bayer Crop Science (formerly Monsanto, St. Louis, MO, USA) provided Cry1A.105 (80% purity, concentration 1mg/mL) and Cry2Ab2 (87% purity, concentration 0.31 mg/mL) in 1 mL aliquots. Cry1A.105 arrived diluted in 25 mM CAPS buffer, pH 10.3 and 1 mM benzamidine-HCl, 0.1 ethylenediaminetetraacetic acid (EDTA) and 0.2 mM dehydrochlorinase (DDT). Cry2Ab2 arrived diluted in 50 mM CAPS buffer, pH 11 and 2 mM DTT. Additionally, Bayer Crop Science also provided both buffers to make the dilutions used in the bioassays. Proteins and buffers were held in a −80 °C freezer.

### 2.4. Bioassays

Bioassays were conducted over the following timeframes: Mexican populations, July 2015 to November 2016; Sus population, June 2014 to February 2017; PR population, June 2016 to February 2017.

Diet overlay bioassays were conducted using the procedure described by Storer et al. [14] using 128-well bioassay trays (Frontier™ Agricultural Sciences), with 1 mL of fall armyworm artificial diet dispensed per well with a surface area of 1.5 cm^2^. Table 1 shows the concentration range used for each population and each protein. A total of 100 μL of each concentration was applied over the diet surface on each well. Two controls were used, which consisted of: (1) diet alone and (2) diet plus the appropriate CAPS buffer. After treatments dried in the wells, one neonate was placed in each well with a small paintbrush. Only healthy, actively moving neonates were chosen for the experiments. Mortality and weight were evaluated seven days after treatment. Larvae that failed to move after prodding, failed to molt to second instar, or weighing ≤0.1 mg were considered dead [16,17,27].

Robertson et al. [32] indicated that 120 individuals in a sample size is sufficient to conduct an insecticide bioassay. For each Bt protein, our work includes between 253–770 individuals. A single repetition consisted of 16 neonates per concentration. Three to five repetitions were conducted per Bt protein with six concentrations covering 0–100% mortality, as determined by previous experiments with diagnostic concentrations. For the PR population, 100% mortality was not achieved with the Cry1F and Cry1Ac proteins.

### 2.5. Statistical Analysis

Mortality data were corrected using Abbott’s formula [33]. Probit analysis [34] was conducted using SAS version 9.3 [35] to calculate values of the slope, the concentration that killed 50% of the larvae (LC_50_), the lethal concentration conferring ninety percent mortality (LC_90_), and fiducial limits (95%) for each population. Resistance ratios were calculated by dividing the LC_50_ values of each field population by the LC_50_ value of the susceptible colony. Log–concentration responses were plotted using Origin^®^ [36].

The effective concentration of each Bt protein that is expected to reduce larval weight by 50% (EC_50_) and confidence intervals (CI) were estimated using a non-linear regression analysis in SAS version 9.335, using the logistic model described by Sims et al. [37]:

Weight = W0/[1+ (dose/EC_50_)b] (1)

## 3. Results

**Figure 1:**
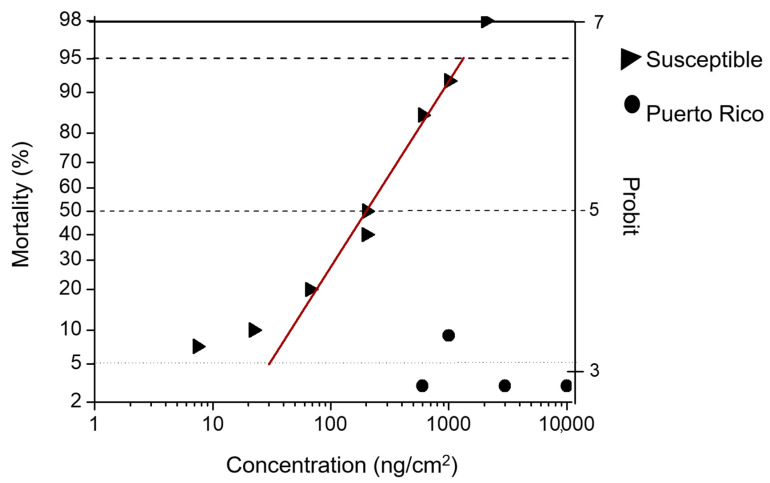
Log concentration-mortality in diet-overlay bioassays to Cry1F. The fall armyworm susceptible laboratory colony (triangles) versus a fall armyworm field population collected in Puerto Rico (circles).

There was a clear difference in Bt protein susceptibility between the Mexican and Puerto Rican field populations (Table 2 and Table 3). The greatest differences were observed in the LC_50_s and EC_50_s for Cry1F and Cry1Ac, with larvae from the Mexican populations needing the lowest concentrations, while larvae from the PR population requiring approximately 12- to 100-fold more (Figure 1, Table 2 and Table 3). Thus, for PR, the LC_50_ and EC_50_ values for Cry1F were close to or greater than 10,000 ng/cm^2^, which was the highest concentration that could be attained with the stock solution.

Even with the highest dose of Cry1Ac, only 62% mortality was achieved in the PR population, so the LC_50_ and EC_50_ values have wide fiducial limits and confidence intervals, respectively. Moreover, the slope value obtained with Cry1Ac in the PR population was significantly lower than the values obtained with the Mexican field populations and the susceptible reference. This indicates that higher protein concentrations are needed in PR fall armyworms to reach a similar mortality response than in the other populations tested. Indeed, this can be observed at the LC_90_ level, where the concentration that is predicted to kill 90% of the population increased 671.8-fold from the concentration estimated for the susceptible reference population.

Reduced weight gain as a sublethal effect of the Bt protein’s activity in the insect’s midgut was measured using the EC_50_ parameter. Therefore, this parameter provides a more sensitive insight into the changes in susceptibility that go beyond the acute toxicity measured by the LC_50_. This is evident in the PR population, where 100% mortality was not achieved with the highest concentration of Cry1Ac, but the reduced weight gain was enough to calculate an EC_50_ value that was 26.7-fold lower than the LC_50_ value. This shows that Cry1Ac still has biological activity against this fall armyworm population from Puerto Rico, yet it is arguable whether this is enough to achieve effective field control. In contrast, fall armyworms from Mexico showed low LC_50_ values with Cry1Ac; in particular, the Sin and Jal populations were statistically more susceptible to this protein than the susceptible reference population, as seen by their non-overlapping fiducial limits.

The Mexican and PR (to a lesser extent) fall armyworm populations were susceptible to Cry1A.105 in comparison with the reference susceptible population at both LC_50_ and EC_50_ levels. Both Jal and Oax were significantly more susceptible to Cry1A.105 than both the susceptible reference and PR populations, since their LC_50_ fiducial limits and EC_50_ confidence intervals do not overlap. PR fall armyworms showed a similar level of susceptibility to the susceptible reference population to Cry1A.105 based on the LC_50_ as observed by their overlapping fiducial limits. However, for the EC_50_, the PR fall armyworms were 2.4-fold less susceptible than the susceptible reference.

For Cry2Ab2, though all populations are susceptible to this protein, the slope value for the susceptible reference is significantly steeper than the one obtained for the field populations. This could indicate that the field fall armyworms tested have a higher degree of variability in their response to this specific protein than the susceptible reference. For the LC_50_, Jal is the only field population we tested with non-overlapping fiducial limits with the susceptible reference. Still, if we look at the EC_50_, both Jal and Oax show lower values with non-overlapping confidence intervals relative to both the susceptible and PR populations, which could mean that these populations are more sensitive to Cry2Ab2.

For the Sin population, 100% mortality was achieved at the lowest concentrations of Cry1A.105 and Cry2Ab2 used (22.2 ng/cm^2^). Therefore, neither LC_50_ nor EC_50_ values were calculated.

Overall, both LC_50_ and EC_50_ values indicate that the fall armyworm populations from Mexico are highly susceptible to all of the Bt proteins tested, while only Cry2Ab2 and Cry1A.105 remain effective against the PR population.

## 4. Discussion

Fall armyworms from Puerto Rico showed resistance to Cry1F and Cry1Ac, while remaining susceptible to Cry1A.105 and Cry2Ab2, despite being exposed to these proteins for several years. Additionally, our study shows that fall armyworm from Mexico displayed low LC_50_ and EC_50_ values to the four purified proteins Cry1F, Cry1Ac, Cry2Ab2, and Cry1A.105. To the best of our knowledge, this is the first official report of the status of fall armyworm susceptibility to Cry1A.105 and Cry2Ab in Mexican field-collected populations.

Previous analysis with Cry1Fa and Cry1Ac with populations collected from the states of Tamaulipas and Estado de Mexico has also shown high susceptibility of fall armyworm to this technology [13]. Furthermore, a recent publication with populations from the Mexican states (Baja California Sur, Chihuahua, Coahuila, Durango, Sinaloa, Sonora, and Tamaulipas) has also reported that fall armyworm is susceptible to Cry1F [38]. Their LC_50_ results in several populations from Sinaloa ranged from 29.4 to 107.2 ng/cm^2^ [38], this is consistent with the results we obtained with our Sin population, displaying an LC_50_ of 29.2 (22.6–36.7) ng/cm^2^.

The first reports of fall armyworm resistance to Cry1F occurred in Puerto Rico [13,14], in populations collected in 2007 and 2008. Our data indicate that fall armyworm from PR are still highly resistant to Cry1F, 10 years after Cry1F expressing Bt maize (TC1507) was removed from the field. Our finding extends the bioassay results by Vélez et al. [39], which show high levels of fall armyworm resistance to Cry1F in PR between 2010 and 2013. Strong selection to Cry1F has been discontinued in Puerto Rico due to the reduced deployment of hybrids containing Cry1F; however, some of the new pyramided Bt corn hybrids that are grown on the island contain Cry1F in combination with other Bt proteins.

Resistance to Cry1F in fall armyworms from Puerto Rico has been described as recessive, conferred by a single, autosomal locus, and with no maternal effects [14,39]. The molecular mechanism of Cry1F resistance in fall armyworm from Puerto Rico and Brazil was characterized as a reduction or complete lack of Cry1F binding to the brush border membrane vesicles (BBMV) in the midgut [31,40]. The authors have proposed that this resistance mechanism is responsible for high levels of cross-resistance to Cry1Aa proteins in populations with field-evolved Cry1F resistance. However, a 2017 study [41] has reported that the field-evolved resistance to Cry1Fa in fall armyworms from Puerto Rico is closely linked to a mutation in an ATP-binding cassette subfamily C2 (ABCC2) gene. This protein has a role as a Cry1Fa receptor in susceptible insects. Low or no cross-resistance has been found to other Cry proteins, such as Cry1Ac, Cry1Ab, and Cry2A [14,31,39,42].

Cry1Ac has been described as having low biological activity against fall armyworm, to the point that it is deemed ineffective [43,44]. Our results show that a Cry1F-resistant fall armyworm from Puerto Rico also displayed a high resistance ratio to Cry1Ac when compared with the susceptible reference. Furthermore, it was demonstrated that Cry1A and Cry1F share midgut-specific binding sites, in both fall armyworm and the European corn borer (*Ostrinia nubilalis*), meaning that cross-resistance between these two proteins might be possible [45]. Although Storer et al. [14] suggested that Cry1Ac and Cry1F do not share the same binding site in the insect’s midgut, the data presented here suggest the opposite. Specifically, our study shows that fall armyworms from the Puerto Rican population are highly resistant to Cry1Ac, despite their lack of prior exposure. On the other hand, fall armyworms from the Mexican populations tested were highly susceptible. The major difference between these populations is prior exposure to Cry1F. Consistent with cross-resistance between Cry1F and Cry1Ac, only fall armyworms from Puerto Rico have a history of Cry1F exposure. Consistent with this observation, an alternative resistance mechanism has been described in fall armyworms from Puerto Rico, a mutation in the receptor SfABCC2 that binds both Cry1Fa/Cry1A [41]. Since this mutation was detected in fall armyworm individuals from Puerto Rico since 2009, resistance to Cry1Ac could be explained as a case of cross-resistance to Cry1F. Screening for the presence of this SfABCC2 mutation in fall armyworms from Ponce, Puerto Rico could help confirm this hypothesis.

Some pyramided Bt corn hybrids containing Cry1A.105 and Cry2Ab2 in addition to Cry1F have been grown in Puerto Rico since the detection of field-evolved resistance to Cry1F [15]. This means that fall armyworm in Puerto Rico has been exposed to these two proteins for at least four years before the present study. Cry1A.105 is a chimeric Bt protein comprised by domain exchange from Cry1Ab (domain I), Cry1Ac1 (domain II), Cry1F (domain III) and Cry1Ac (C-terminal) [46]. Thus, some level of cross-resistance between Cry1A.105 and Cry1F proteins can be expected. A case of cross-resistance between Cry1F and Cry1A.105 in fall armyworm was already reported from Brazil [28]. Insects from the Brazilian population that were collected from the field, and further selected for Cry1F resistance, showed considerable levels of cross-resistance to Cry1A.105 and Cry1Ab, but only low levels of resistance to Cry2Ab2 [28]. Similarly, selecting for Cry1A.105 resistance in fall armyworm resulted in high levels of cross resistance to Cry1 proteins [47].

In this study, the fall armyworm population from Puerto Rico remained largely susceptible to Cry1A.105, despite being resistant to Cry1F and Cry1Ac. These results are in contrast with the aforementioned reports of significant cross-resistance between these proteins in Brazil and the United States [28,47]. However, considering the EC_50_ parameter, insects from the PR population were 2.4-fold less susceptible to this protein than the susceptible reference population. This result could indicate that susceptibility to Cry1A.105 is decreasing in this fall armyworm population, or that resistance to Cry1F and Cry1Ac confers low levels of cross-resistance to Cry1A.105. A potential reason for low or no cross-resistance between Cry1F and Cry1A.105 may be the source of the resistant population, with different resistant alleles being present in different regions. In addition, we used fall armyworms that evolved resistance in the field after exposure to Cry1F, while in the referenced studies insects were lab-selected for Cry1F resistance. This could also have selected for different resistance mechanisms in the different populations.

Similarly, our fall armyworm populations from Mexico and Puerto Rico were highly susceptible to Cry2Ab2. Lower susceptibility to Cry2Ab2 protein could be anticipated in fall armyworms from Puerto Rico. However, midgut binding experiments show that Cry2Ab does not share binding sites with Cry1A and Cry1F proteins in *Heliothis virescens*, *Helicoverpa zea*, *Helicoverpa armigera*, *Helicoverpa puntigera* [48,49], or fall armyworm [45]. Furthermore, though the laboratory-selected Cry1F resistant fall armyworm population from Brazil showed low levels of resistance to Cry2Ab2 (10-fold), cross-resistance was ruled out by using plant bioassays [28].

Our results suggest that Cry1F and Cry1Ac resistance is established in fall armyworm from Puerto Rico. Additionally, the Puerto Rican fall armyworm population used in this work also displayed high levels of field-evolved resistance to five insecticide modes of action [12]. The resistance to Cry1F and Cry1Ac reported in this study expands on the critical status of fall armyworm management on this island, suggesting that a general mechanism of resistance might be at play in this population. On a brighter note, fall armyworm management in Puerto Rico has successfully preserved susceptibility to Cry2Ab2 and Cry1A.105 proteins to date.

The exceptional migratory capacity that fall armyworm exhibits may play a crucial role in the spread of the resistance alleles to other regions. A study analyzing mitochondrial haplotype ratios has concluded that fall armyworms from Puerto Rico showed more similarities with fall armyworm from Florida (suggesting migrations from Puerto Rico to Florida) than those from Texas and Brazil [50]. The detection of Cry1F-resistant fall armyworm in Florida in 2012–2013 [19] appears to confirm this interaction and raises concerns about the spread of Bt resistance alleles into other regions where this pest is already a management challenge. Yet, the mutation in the SfABCC2 gene conferring resistance to Cry1F in fall armyworms from Puerto Rico has not been detected in fall armyworms from Florida [41], suggesting limited migration from Puerto Rico to the US mainland.

Furthermore, it was determined that there is limited genetic interactions between fall armyworms from North and South America in the Lesser Antilles [51]. Fall armyworm movements in the Caribbean are driven by two different wind transport systems, which hinder the probability that populations located in the two hemispheres may coincide within this region [51]. In addition, fall armyworms from four Mexican states collected between 2013 and 2014 were characterized comparing amplified fragment length polymorphisms (AFLP) and haplotype ratios with populations from Texas and South America (Argentina and Brazil) [52]. The study has proposed that fall armyworms from Mexico have relatively low genetic interactions with populations from the rest of the hemisphere. This could mean that migration of resistance alleles from mainland US, Puerto Rico and South America to Mexico poses a lower risk for the evolution of resistance to Bt proteins in fall armyworms from Mexico. The extreme susceptibility to Bt Cry proteins in the populations tested in this study suggests that Bt corn has high potential for fall armyworm control in Mexico. This country may benefit from research that has been generated regarding resistance management to Bt technology, and from the lessons learned from regions where this pest has developed field-evolved resistance to Bt crops. However, this technology should not be seen as a single solution for pest problems; in addition to high dose/refuge strategy for resistance management, IPM should include other control tactics, such as crop rotation and fallow periods. The fact that our Mexican populations were highly susceptible to Bt proteins seems to indicate that they have not interacted with resistant populations from Puerto Rico or Florida. However, the limitation of this study is that we only worked with four populations from regions that were very far away from each other in Mexico, and there is a possibility that fall armyworms from other Mexican regions have already come in contact with fall armyworms carrying resistance alleles.

Monitoring for susceptibility to current control tools is especially important in regions where fall armyworm is a novel pest. Such is the case in several countries in Africa, Asia, and more recently in Australia, where fall armyworm’s presence was officially confirmed in 2016, 2018, and 2019, respectively [6,7]. A recent study has determined that the invasive fall armyworms from six African countries likely originated from Florida and the Greater Antilles [53]. This means that fall armyworms invading Africa and Asia have already been exposed to different classes of pesticides, though specifics are yet unknown. Moreover, there have already been reports of inadequate fall armyworm control with pyrethroid insecticides in Africa [54]. While new management strategies are being designed for regional implementation, such as push–pull technology [55], farmers are still using conventional insecticides to control fall armyworm infestations [54,56]. For instance, a study from Ghana and Zambia has shown that chemical control of fall armyworm has been adopted by approximately 50% of small holder farms, mainly using pyrethroids, organophosphates and glutamate-gated chloride channel allosteric modulators [56]. Synthetic pesticides can be an expensive management tool for low income families, and most mechanic or cultural control alternatives are time and labor consuming.

GE technology might be more suitable for large-scale farmers and some small scale-farmers; however, its adoption can help reduce synthetic insecticide applications to control fall armyworm, and the efficacy of the available active ingredients can have a longer lifespan. The lessons learned from the development of Cry1F resistance in Puerto Rico, Brazil, and Argentina have recently been summarized by Huang et al. [57]. The top three factors involved in the development of Cry1F resistance were: (1) deployment of single toxins, (2) cross-resistance in fall armyworm to Cry1 proteins, and (3) Bt crops did not express high doses of the Bt proteins [57]. This highlights the need to consider these factors in future deployment of Bt toxins and determine the best strategies to incorporate resistance management plans and different actors including academia, growers, industry and regulatory agencies [58].

Our work confirms the stability of the resistance to Cry1F in fall armyworm populations from Puerto Rico. This is consistent with previous reports that indicate a lack of strong fitness costs associated with resistance to Cry1F in different populations from this island [27,42]. Additionally, an extensive work characterizing Cry1F resistance in fall armyworms from Brazil [59] has determined that resistance to this Bt protein can be due to the interaction of several resistant alleles, and can develop independently in different populations in the presence of selection pressure. This highlights the importance of studying resistance case by case, even with the same species, since resistance may evolve through different mechanisms.

## 5. Conclusions

In conclusion, this is a unique research study that shows the stability of resistance of fall armyworm from Puerto Rico to Cry1F and resistance to Cry1Ac, but the lack of cross-resistance to Cry1A.105 and Cry2Ab2. Susceptibility to all Bt proteins was found in the Mexican populations. Therefore, it is crucial to monitor the susceptibility of target pests to GE technology; any changes towards resistance development could be better addressed and understood when detected early.

## Figures and Tables

**Table 1:** *Bacillus thuringiensis* (Bt) protein concentrations used in concentration–mortality response bioassays.

Population	Bt Protein	Concentration Range (ng/cm^2^)
Susceptible	Cry2Ab2	10–1000
	Cry1A.105	10–1000
	Cry1F	7.4–1000
	Cry1Ac	7.4–1300
Jalisco Oaxaca	Cry2Ab2	0.7–200
	Cry1A.105	0.02–67
	Cry1F	7.4–1000
	Cry1Ac	7.4–1000
Sinaloa	Cry2Ab2	22.2–1300
	Cry1A.105	22.2–1300
	Cry1F	7.4–1000
	Cry1Ac	22.2–1300
Puerto Rico	Cry2Ab2	7.4–6000
	Cry1A.105	7.4–6000
	Cry1F	22.2–10,000
	Cry1Ac	22.2–6000

**Table 2:** Concentration–mortality response (ng/cm^2^) of fall armyworm from several Mexican states and Puerto Rico to Bt proteins.

Bt Protein	Pop ^a^	n ^b^	LC_50_^c^	95% FL ^d^	RR_50_^e^	LC_90_^c^	95% FL ^d^	RR_90_^e^	Slope ± SE ^f^	X^2 g^
Cry1F	SUS	338	174.4	135.7, 222.6	1.0	849	606.6, 1349	1.0	1.9 ± 0.2	0.9
	SIN	624	29.2	22.6, 36.7	0.2	201	145, 309	0.2	1.5 ± 0.1	2.8
	JAL	384	42.8	29.8, 59.8	0.2	463	275.4, 1015	0.5	1.2 ± 0.2	4.3
	OAX	384	26.5	0.03, 122	0.2	502	113, 1.92 × 10^12^	0.6	1 ± 0.3	2.5
	PR	256	>10,000	Ne ^h^	ne	>10,000	ne	ne	ne	ne
Cry1Ac	SUS	770	148.2	120, 183.2	1.0	1050	750.3, 1635	1.0	1.5 ± 0.1	4.0
	SIN	512	15.3	8, 22	0.1	78	57, 125	0.1	1.8 ± 0.3	0.3
	JAL	380	34.2	20.5, 52.3	0.2	817.7	397.9, 2781	0.8	0.9 ± 0.1	1.5
	OAX	385	188.8	132, 280	1.3	3262	1669, 8983	3.1	1 ± 0.1	3.4
	PR	624	1815	934.5, 5374	12.2	705,419	95,917, >100,000	671.8	0.5 ± 0.08	1.4
Cry1A.105	SUS	641	201.9	150.4, 266.3	1	859	586.6, 1576	1	2 ± 0.3	4.5
	SIN	383	ne	ne	ne	ne	ne	ne	ne	ne
	JAL	256	4.6	3.2, 6.4	0.02	28.3	17.8, 57.5	0.03	1.6 ± 0.2	4.5
	OAX	560	14.5	3.4, 139.7	0.07	164.4	37.8, >100,000	0.2	1.2 ± 0.3	5.8
	PR	303	273.8	172.5, 442.8	1.4	3234	1542, 12,547	3.8	1.2 ± 0.2	1.4
Cry2Ab2	SUS	640	173.2	130.8, 214.1	1	469	373.5, 649.9	1	3 ± 0.4	5
	SIN	381	ne	ne	ne	ne	ne	ne	ne	ne
	JAL	256	49.7	30.3, 94	0.2	1040	398.9, 5369	2.7	0.9 ± 0.1	3.2
	OAX	410	13.3	3.1, 165.5	0.1	1762	150.3, >100,000	4.6	0.6 ± 0.2	2.4
	PR	253	119.2	68.5, 187	0.5	2092	1091, 6065	5.5	1 ± 0.2	0.4

^a^ Fall armyworm population, ^b^ total number of insects used, ^c^ lethal concentration (LC) expressed in nanograms of active ingredient per square centimeter of diet (ng/cm^2^), ^d^ fiducial limits, ^e^ resistance ratio = LC of fall armyworm field population over the LC of the susceptible reference population, ^f^ probit slope ± standard error (SE), ^g^ chi-square value, ^h^ ne = not estimated due to insufficient response to treatments.

**Table 3:** Effective concentration of Bt proteins that is expected to reduce weight by 50% (ng/cm^2^) in fall armyworm from several Mexican states and Puerto Rico.

Bt Protein	Pop ^a^	n ^b^	EC50 ^c^	SE ^d^	95% CI ^e^
Cry1F	SUS	338	11	1.3	8.2, 13.4
	SIN	624	16	1.4	12.5, 18.7
	JAL	384	5	1.2	2.3, 7.4
	OAX	384	7	0.8	5.2, 8.8
	PR	256	>10,000	ne	ne
Cry1Ac	SUS	770	5	1.1	2.4, 6.9
	SIN	512	23	0.2	22.1, 23.5
	JAL	380	1.6	0.6	0.4, 2.8
	OAX	385	3	0.6	1.5, 4
	PR	624	68	10.8	43, 92
Cry1A.105	SUS	641	31	5	19.6, 42.8
	SIN	383	ne	ne	ne
	JAL	256	1.3	0.2	0.9, 1.8
	OAX	560	1.4	0.3	0.8, 2.2
	PR	303	73.1	9.3	51.7, 94.4
Cry2Ab2	SUS	640	111	27	52.4, 169.6
	SIN	381	ne	ne	ne
	JAL	256	5.4	1.4	2.3, 8.5
	OAX	410	1.1	0.2	0.7, 1.6
	PR	253	24.7	5.8	11.7, 37.7

^a^ Fall armyworm population, ^b^ total number of insects used, ^c^ effective concentration of Bt protein that is expected to reduce larval weight by 50% expressed in nanograms of active ingredient per square centimeter of diet (ng/cm^2^), ^d^ standard error, ^e^ confidence intervals, ne = not estimated due to insufficient response to treatments.
